# Oncologic Outcomes of Interventions to Decrease Allograft Ischemia-Reperfusion Injury within Patients Undergoing Liver Transplantation for Hepatocellular Carcinoma: A Systematic Review

**DOI:** 10.3390/curroncol31060221

**Published:** 2024-05-21

**Authors:** Matheus D. Faleiro, Zuhaib M. Mir, Yara Azizieh, Stephanie E. Hiebert, Scott M. Livingstone, Mark J. Walsh, Boris L. Gala-Lopez

**Affiliations:** 1 Faculty of Medicine, Federal University of Minas Gerais, Belo Horizonte 31270-901, MG, Brazil; 2 Department of Surgery, Dalhousie University, Halifax, NS B3H 4R2, Canada; 3 Department of Pathology, Dalhousie University, Halifax, NS B3H 4R2, Canada; 4 Beatrice Hunter Cancer Research Institute, Halifax, NS B3H 0A2, Canada

**Keywords:** hepatocellular carcinoma, liver transplant, cancer recurrence, ischemia-reperfusion injury, systematic review

## Abstract

Ischemia-reperfusion injury (IRI) during liver transplantation has been implicated in the recurrence of hepatocellular carcinoma (HCC). This systematic review aimed to evaluate interventions to reduce IRI during liver transplantation for HCC and their impact on oncologic outcomes. A comprehensive literature search retrieved four retrospective studies involving 938 HCC patients, utilising interventions such as post-operative prostaglandin administration, hypothermic machine perfusion, and normothermic machine perfusion. Overall, treated patients exhibited reduced post-operative hepatocellular injury and inflammation and significantly enhanced recurrence-free survival. Despite these promising results, the impact of these interventions on overall survival remains unclear. This underscores the imperative for further prospective research to comprehensively understand the efficacy of these interventions in HCC patients undergoing transplantation. The findings highlight the potential benefits of these strategies while emphasising the need for continued investigation into their overall impact.

## 1. Introduction

Liver transplantation (LT) is the treatment of choice for numerous end-stage liver diseases, with hepatocellular carcinoma (HCC) being the main indication for LT in the USA [1]. LT is the treatment of choice for HCC because it eliminates the tumour and removes the compromised liver parenchyma, which has limited functional capacity and is susceptible to carcinogenesis [2]. The Milan criteria, which consider the presence of a tumour of 5 cm or less in diameter in patients with single HCC and no more than three tumour nodules, each 3 cm or less in diameter, in patients with multiple tumours, were the first patient selection criteria established to define the optimum disease burden for which liver transplants can achieve excellent long-term outcomes [2]. These criteria were subsequently expanded on by the University of California San Francisco (UCSF) criteria, which has shown excellent post-transplant outcomes, considering patients with a single nodule up to 6.5 cm or up to three lesions, with the largest one having a size of 4.5 cm or more, with the combined sum of their diameters being 8 cm or greater [3,4,5,6]. 

Despite this well-established patient selection criteria, HCC recurrence after LT remains a significant concern, being reported in up to 70% of cases at five years, reflecting either intrahepatic metastases or the development of de novo tumours [7]. Recurrence after LT can be estimated by the Risk Estimation of Tumour Recurrence After Transplant (RETREAT) score, which holds significant value in guiding post-LT surveillance and treatment by utilising pre-transplant alpha-fetoprotein (AFP) levels and pathological explant data [8]. Recurrence could be associated with tumour-related factors (e.g., size, number of nodules, differentiation, and oncological markers), as well as patient factors (age, comorbidity, liver function, possible viral load, presence and activity of hepatitis, presence, and activity of liver cirrhosis), and treatment factors (type of treatment, margins, and characteristics of resection) [9]. 

Due to its surgical mechanism, LT results in ischemia-reperfusion injury (IRI), defined as tissue injury that occurs when the blood supply to organs is interrupted and re-established after several hours. This event triggers reactive oxygen species production and an inflammatory response that causes additional tissue damage. IRI in the liver has been linked to HCC recurrence after LT, with several proposed mechanisms. First, IRI-induced damage to the hepatic microenvironment disrupts microvascular function, leading to hypoxia and the activation of the HIF-1α pathway, which supports tumour cell survival and growth. Additionally, IRI-induced inflammation promotes the expression of adhesion molecules on vascular endothelium, facilitating the attachment of circulating tumour cells and their subsequent transmigration. IRI also affects tumour cell invasion and migration through modulation of cell motility regulators and the activation of the CXCL-10 pathway, which causes the recruitment of progenitor cells, creates a pro-angiogenic environment, and suppresses immune responses against tumours. Several preclinical studies have indicated that IRI is related to HCC recurrence after LT, and these data have been further endorsed by recent clinical evidence [10,11,12]. In this context, novel strategies to reduce IRI and consequently minimise HCC recurrence after LT are needed. 

This systematic review aims to synthesise and evaluate the evidence related to interventions to decrease allograft IRI during LT in patients with HCC and the subsequent effect on cancer recurrence and cancer-specific survival. This review will provide an overview of the strategies currently studied for reducing IRI and summarise the main oncological outcomes associated with each technique. 

## 2. Materials and Methods

### 2.1. Study Design 

A systematic review of the literature was conducted to identify clinical studies that had utilised strategies to reduce IRI in grafts retrieved for LT in patients with HCC. The review was designed and reported in accordance with the preferred reporting items for systematic reviews and meta-analyses (PRISMA) guidelines [13]. We did not register our systematic review on the PRISMA website, but we did register it on the PROSPERO website under the ID CRD42023459207.

### 2.2. Search Strategy 

A comprehensive literature search was conducted to identify published studies on PubMed, Ovid MEDLINE, and EMBASE platforms. All databases were searched from their inception until October 2023 without language or date restrictions. The attached Supplementary Table S1 presents the complete search strategies for all databases. Cited references were also examined to ensure the saturation of the literature. The records obtained from the databases were uploaded into Covidence systematic review software for screening (https://www.covidence.org/; accessed on 14 February 2024).

### 2.3. Inclusion and Exclusion Criteria

Prospective and retrospective studies of LT for HCC in adult patients were included in a comparative analysis of any clinical intervention aimed at reducing IRI in the grafts and assessed tumour recurrence during their follow-up period. Studies were excluded if they used animal models of IRI to assess oncologic outcomes, if their outcomes did not include HCC recurrence or survival, or if they were duplicate studies not initially screened out. Furthermore, narrative literature reviews and studies with no patient outcomes were also excluded.

### 2.4. Screening and Data Collection

Screening of all studies for inclusion was conducted independently by two reviewers (MDF and ZMM). Any discrepancies were resolved via discussion and consensus between the reviewers. Data abstraction from the final set of included studies was carried out independently by both reviewers in duplicate using a standardised data extraction table developed a priori.

### 2.5. Qualitative Assessment 

The qualitative assessment of included studies was performed using the Cochrane risk of bias in non-randomised studies of interventions (ROBINS-I) tool. Both reviewers independently assessed each study for risk of bias, and any disagreements were resolved via discussion and consensus between the reviewers. 

## 3. Results

### 3.1. Studies and Patient Characteristics 

**Figure 1:**
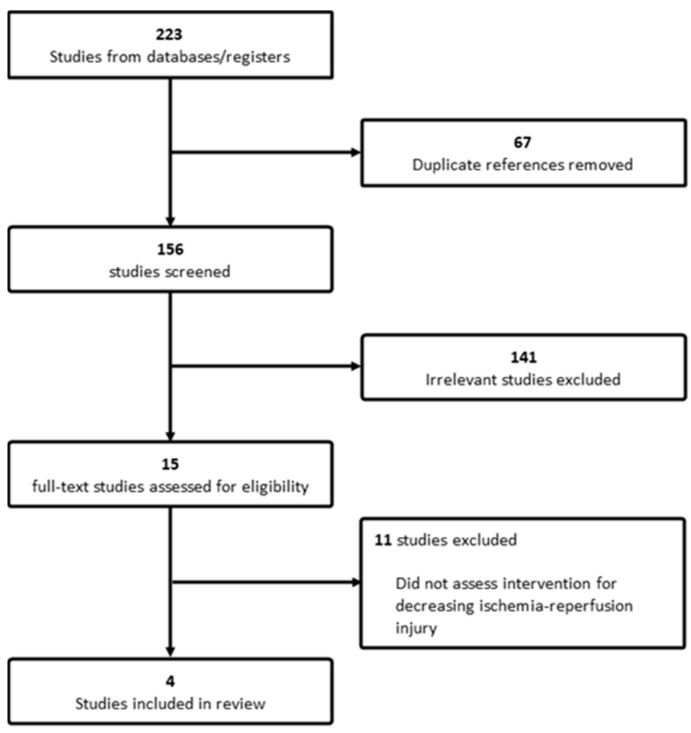
Flow diagram of study selection for evidence synthesis.

Our search results yielded 223 studies for screening, and after removing 67 duplicates, two independent reviewers screened the remaining 156 abstracts for relevance, identifying 15 studies for full-text assessment. Four studies met the inclusion criteria and were incorporated into this review. The flow diagram of study selection for evidence synthesis is presented in Figure 1. All the studies in this review had a retrospective design. The earliest one, a single-centre study conducted in Germany, was published in 2015 by Kornberg et al. Mueller et al. (2020) reported a multicentre study from two institutions in Switzerland [14,15]. The remaining two studies were published by Tang et al. (2021) and Rigo et al. (2023) in centres in China and Italy, respectively [16,17]. A summary of the studies included in the literature can be found in Table 1. 

Overall, 938 patients who underwent LT for HCC were analysed, with 239 receiving an intervention to reduce IRI on the hepatic allograft before the surgical procedure. The Milan criteria were adopted to determine patient eligibility for LT in three of the included studies, although one study (Rigo 2023) adopted the Metro ticket 2.0 [17]. The Model for End-Stage Liver Disease (MELD) score was utilised as a prognostic score by three studies, while Kornberg et al. (2015) adopted the Child–Pugh classification [14]. 

Tumour characteristics were also reported among the included papers, with the size of the largest lesion varying between 32 mm (18.5–53.5 mm) and 60 mm (10–180 mm) (median and range, respectively). Additionally, microvascular invasion was observed in 47 patients within the intervention group. Transplant candidates of two studies underwent neoadjuvant locoregional cancer therapy (LRCT) before transplantation, mainly performed by transarterial chemoembolisation in Mueller 2020 [15].

### 3.2. Interventions to Decrease Ischemia-Reperfusion Injury 

Across all included studies, sample sizes in the intervention group ranged between 30 and 80 patients. The interventions applied included post-operative administration of the prostaglandin E1 (PGE1) analogue Alprostadil in one study, normothermic machine perfusion (NMP) in one study, and hypothermic machine perfusion (HMP) in two studies. The paper using Alprostadil reported that the therapy was started post-operatively in the intensive care unit and maintained at a minimum until patients were transferred to a regular ward for up to 7 days post-LT. The drug was administered by a continuous infusion, beginning at a low dose (0.1 mg/kg/h) and slowly increasing to a maximum dose (0.5 mg/kg/h). In this study, hemodynamic instabilities, thrombocytopenia, bleeding, or allergic reactions were considered relevant adverse effects and led to a stepwise taper or complete withdrawal of the treatment [14]. 

NMP was adopted in one study by using the Liver Assist device (Organ Assist, Groningen, The Netherlands). To conduct an ischemia-free liver transplant, initially, the authors utilised an in situ normothermic mechanical perfusion followed by ex situ NMP [16]. Details regarding the composition of the perfusate used in this study are presented in Table 1. The NMP was interrupted only after the native blood supply from the recipient’s portal vein and hepatic artery to the graft was re-established, ensuring that the allograft did not suffer ischemic damage. 

The utilisation of HMP was reported by two studies, each with some differences. Both Mueller et al. (2020) and Rigo et al. (2023) described the adoption of static cold storage before machine perfusion [15,17]. However, Mueller et al. (2023) employed hypothermic oxygenated perfusion (HOPE), while Rigo et al. (2023) used dual-hypothermic oxygenated machine perfusion (D-HOPE) [15,17]. Mueller reported no information regarding the perfusion technique (2020) [15]. Rigo et al. (2023) reported the utilisation of the LiverAssist device (XVIVO, Goteborg, Sweden) and the Belzer MP solution (BridgeToLife, Northbrook, IL, USA) as perfusate [17]. The perfusion was maintained at a settled pressure of 3–5 mmHg in the portal vein and 25 mmHg in the hepatic artery during a minimum period of 90 min during recipient hepatectomy [17]. The graft implantation was performed after the interruption of D-HOPE, and the graft was flushed with chilled 5% albumin before implantation into the recipient [17]. 

### 3.3. Immunosuppressive Regimen and Rejection Incidence 

Regarding the immunosuppressive regimen, both Rigo 2023 and Tang 2021 included basiliximab as an induction therapy [16,17]. In Tang 2021, Rigo 2023, and Kornberg 2015 studies, a calcineurin inhibitor and mycophenolate mofetil were administered for immunosuppressive maintenance therapy [14,16,17]. In contrast, Mueller 2020 reported the use of tapered steroids and tacrolimus monotherapy, with the addition of basiliximab in recipients with impaired kidney function after liver transplantation [15]. The immunosuppressive regimens were generally comparable between the intervention and control groups in the included studies, except for Rigo 2023, which indicated a higher frequency of basiliximab administration as induction therapy in the intervention group [17]. 

Rejection episodes were analysed in two of the included studies. Rigo 2023 reported similar results when comparing the early and late incidence of rejection episodes between its intervention and control groups. In contrast, Mueller 2020 found a significantly lower acute rejection rate in its intervention group [15,17]. 

### 3.4. Post-Operative Outcomes 

Table 2 summarises peri-operative and oncologic outcomes of strategies to decrease IRI within the included studies [14,15,16,17]. Overall, treated patients showed significantly lower levels of markers of hepatocellular injury and inflammation in the immediate post-operative period. Lower serum levels of ALT (Tang 2021, Rigo 2023, Kornberg 2015) and AST (Tang 2021, Rigo 2023, Kornberg 2015) were reported in the included studies [14,16,17]. Mueller 2020 indicated a different result, with recipients of DCD livers experiencing higher levels of ALT on day one after LT [15]. Post-transplant C-reactive protein (CRP) levels were also found to be lower in the intervention group of two of the included studies (Kornberg 2015, Mueller 2020) [11,15]. A lower incidence of early allograft dysfunction (EAD) was also reported in the intervention group of one of the included studies (Tang 2021) [16]. 

Consistently, significantly higher recurrence-free survival (RFS) was reported in the intervention group across the included studies compared to their control groups. The 1-year RFS was found to be 92.2% (Tang 2021), 96% (Rigo 2023), and 100% (Mueller 2020); the 3-year RFS was found to be 86.7% (Tang 2021) and 87.9% (Kornberg 2015); 5-year RFS was found to be 92% (Mueller 2020) and 85.7% (Kornberg 2015) [14,15,16,17]. 

Three included studies analysed overall survival in their cohorts, with conflicting survival results [14,16,17]. Tang et al. (2021) reported overall survival rates at 1 and 3 years in their intervention group as 96.7% and 90.6%, respectively, higher than those in the control group. However, these differences were not statistically significant [16]. On the other hand, Kornberg et al. (2015) suggested a survival advantage in their intervention group, with overall survival rates at 3 and 5 years of 91.5% and 82.8%, respectively, compared to 74.5% and 65.7% in the control group. Rigo et al. (2023) reported an estimated 5-year survival of 90% in both the intervention and control group, according to the Metroticket 2.0 model, which suggests a comparable 5-year survival probability by preservation modality [14,17]. 

### 3.5. Risk of Bias 

The risk of bias assessments for the included studies are presented in Table 3. In general, all the studies demonstrated a moderate risk of bias, primarily due to confounding and participant selection. Universally, all studies assessed were deemed to have a moderate risk of bias due to confounding, mainly attributable to the methods employed for mitigating potential confounding factors. Three studies presented a moderate risk of bias in selecting participants, due to the selection of participants in the analysis based on participant characteristics observed after the intervention. 

## 4. Discussion

We conducted a systematic review to synthesise and evaluate the evidence relating to interventions to reduce IRI during LT in patients with HCC. We identified three interventions from the literature, including a PGE1 analogue administration, HMP, and NMP. In all included studies, treated patients exhibited significantly reduced hepatocellular injury and a markedly higher rate of RFS than their respective control groups. This suggests a reduced impact caused by IRI on hepatic parenchyma. Additionally, we observed a lower frequency of rejection episodes in the groups that underwent treatments aimed at mitigating IRI. Our findings are significant because previous studies have established a link between the IRI during LT and the recurrence of HCC. Therefore, reducing the extension of IRI could potentially contribute to better preservation of hepatic grafts and improved outcomes following LT. 

IRI is an inevitable aspect of organ transplantation and plays a pivotal role in the adverse outcomes of liver transplantation, making it responsible for approximately 10% of early liver graft failures [18,19]. The temporary halt of blood flow during transplantation leads to ischemia, causing liver tissue damage due to oxygen and nutrient deprivation. Subsequent reperfusion aggravates the damage through inflammatory responses and oxidative stress [20]. The role of IRI in LT has also been linked to HCC recurrence [11,12,21]. During LT, graft manipulation may inadvertently disperse cancer cells into the systemic circulation [22]. Clinical studies have shown that when coupled with the hypoxic effects of IRI, an inflammatory microenvironment is created that fosters the proliferation of cancer cells [22]. Moreover, releasing damage-associated molecular patterns (DAMPs), cytokines, and adhesion molecules in response to IRI initiates downstream pathways responsible for angiogenesis. This angiogenesis can nourish existing cancer cells and, consequently, encourage the growth of residual cancer cells [22]. 

IRI is even more concerning considering the utilisation of expanded criteria donor (ECD) grafts. These grafts encompass donations from individuals with prior steatosis, malignancies, viral infections, as well as elderly donors, donors after cardiac death, and others whose utilisation has been on the rise in recent years to expand the donor pool [23]. Given the state of ECD grafts, they often carry pre-existing damage and have been thought to exhibit heightened susceptibility to IRI due to multiple factors, including compromised microcirculation, elevated vulnerability to lipid peroxidation, intensified neutrophil activity, an elevated level of endoplasmic reticulum stress and, in the case of HCC, a higher recurrence rate from DCD grafts [23]. In this systematic review, Mueller et al. (2020) [15] reported the usage of DCD grafts, while Rigo et al. (2023) [17] described the usage of both DCD and extended criteria DBD grafts. Rigo et al. (2023) [17] further highlighted a preferential use of D-HOPE in grafts from ECD. This decision aligns with a recent randomised controlled trial, indicating that HOPE, compared to SCS, significantly reduces early allograft injury and improves post-transplant outcomes in ECD-DBD liver transplantation [24]. 

Ex vivo organ preservation has recently re-emerged due to its potential protective effects on marginal grafts from ECD [25]. Machine preservation of grafts offers several modalities with variations in temperature and oxygenation, including hypothermic, normothermic, and sub-normothermic settings. Clinically, hypothermic and normothermic preservation have been explored the most and were the interventions used to lower ischemic damage in three studies included in this systematic review. 

Hypothermic machine perfusion maintains grafts between 4 and 12 °C to reduce metabolism while ensuring a continuous circulation of preservation solution throughout the graft, which helps wash out waste accumulated during preservation [26]. In addition to minimising IRI, hypothermic machine perfusion has been found to reduce rates of early graft dysfunction and biliary complications compared to the preservation gold standard, static cold storage [26,27]. 

Hypothermic oxygenated machine perfusion is a modification of hypothermic machine perfusion that introduces oxygenation to remove metabolic waste further, prevent the depletion of ATP, and reduce IRI. This modality perfuses the portal vein but can also be performed through the portal vein and hepatic artery, known as dual-hypothermic oxygenated machine perfusion [28,29]. Mueller and colleagues used hypothermic oxygenated machine perfusion to decrease IRI due to HCC to prevent tumour recurrence successfully. A similar study conducted by Rigo and colleagues using dual-hypothermic oxygenated machine perfusion as an intervention to decrease HCC recurrence showed different results. Despite studies showing dual-hypothermic oxygenated machine perfusion’s ability to reduce IRI, this study had comparable results to the control regarding HCC recurrence and early rejection rates, which could be attributed to the sample size. Further research comparing the difference in the ability of hypothermic oxygenated machine perfusion and dual-hypothermic oxygenated machine perfusion to reduce IRI is needed to understand the effects of MP in reducing HCC recurrence. 

NMP is another modality of machine perfusion currently adopted in clinical practice. It is particularly promising because it mimics the physiological characteristics of the liver, maintaining the graft by providing oxygen, nutrients, and other vasoactive drugs at a temperature of 37 °C. This results in a better energetic balance for liver cells and fewer IRI mediators [30]. In a recent meta-analysis of randomised controlled trials, NMP was associated with reduced liver injury and reduced biliary complications [31,32]. When compared to HMP, it is crucial to notice that NMP allows reduced periods of static cold storage (SCS) or even the complete elimination of SCS entirely, termed “ischemia-free liver transplantation” (IFLT), which was adopted as a strategy to reduce IRI during LT by Tang et al. (2021) [16]. In a previous meta-analysis comparing NMP and HOPE/D-HOPE, adopting the hypothermic technique had been associated with improved graft and patient survival and reduced biliary complications. A similar trend was noticed in this work when comparing 1-year RFS between these two modalities of MP, with the papers that adopted HOPE/D-HOPE having a higher 1-year RFS than the paper that adopted NMP. However, new research is needed to understand the effects of MP on RFS [16]. 

Prostaglandins are a group of compounds primarily synthesised from arachidonic acid through the cyclooxygenase pathway. The liver is the primary organ in synthesising these molecules, which are significantly released by activated Kupfer cells during organ reperfusion and play a crucial role in offering various protective effects on free oxygen radicals, sinusoidal endothelium, and platelets [33]. In the liver, prostaglandins confer their protective effects on IRI-damaged tissue mainly by inhibiting the generation of reactive oxygen species, preventing leukocyte migration, reducing the synthesis or production of membrane degradation products, improving hepatic insulin and lipid metabolism, and regulating the production of inflammatory cytokines and cell adhesion molecules [34]. The interaction between these anti-oxidative and anti-inflammatory effects potentially contributes to limiting the damage caused by IRI to the hepatic parenchyma, which could explain the reduced risk of early tumour recurrence found by Kornberg et al. (2015) in 59 patients treated with the administration of the stable PGE1 analogue Alprostadil [14]. This finding could be promising in clinical settings, as the administration of Alprostadil could potentially be integrated into post-transplant approaches to mitigate oncological risks in patients with advanced HCC. 

Besides the pharmacological and technological strategies cited in this paper to decrease IRI, changes in surgical intervention could also play an essential role in avoiding ischemia. Ischemic preconditioning is a well-established approach for lessening reperfusion injury. It consists of brief intermittent periods of ischemia and reperfusion, achieved by the portal triad occlusion, followed by a period of normothermic reperfusion [35]. This approach has been presented to induce a state of protection against sustained ischemic insult in multiple clinical studies, and although the precise mechanisms by which this approach confers protection against IRI are not fully understood, incorporating this intra-operative strategy must be considered in clinical practice. Its effectiveness could be enhanced by integrating it with other methods to avoid IRI, as cited in this paper [36]. 

The financial implications of integrating these innovative technologies into clinical practice need further evaluation. Although the initial expenses for such technologies typically decrease as they become more widely used and as the market becomes more competitive, it is hypothesised that adopting these technologies in current clinical practice could potentially lower overall transplant process and patient care costs, mainly due to the technologies’ ability to reduce recurrence episodes, which pose a financial burden to healthcare providers due to the necessity of surgical reapproach. Regarding the cost advantage of MP, some early evidence exists regarding the potential economic benefit of this technology, mainly due to lower post-operative complications and shorter hospital stay associated with livers preserved by MP [37]. Despite the lack of studies aimed at assessing the cost burden associated with the incorporation of explicitly addressing the financial impact of incorporating PGE1-analogue drugs into post-transplant management protocols, it is suggested that this strategy may also lead to cost reductions, mainly related to the increase in RFS and the decrease in the reapproach necessity. However, new studies focused on the cost-effectiveness assessment of these technologies are further needed. 

This work has limitations. First, we could not conduct a quantitative synthesis of results due to the heterogeneity of patients and interventions, indications for LT, and outcome definitions across the studies. Additionally, the role that the strategies to prevent IRI could play in overall patient survival could not be fully comprehended in this systematic review because this outcome was reported by only two of the included studies, and their results were conflicting. Despite these limitations, this study employed a comprehensive search strategy to conduct the first systematic review of strategies to reduce IRI during LT for HCC, providing an essential overview of this body of literature. The strengths of our study include the search strategy that was utilised to conduct a detailed analysis with minimal restrictions, using validated and sensitive search strings to identify the included studies. In future directions, new clinical studies are needed to compare the different interventions adopted to avoid IRI in this paper, with more extended follow-up periods and more patients. Reports on overall graft and patient survival, RFS, and rejection episodes should be encouraged in these novel studies, which will help us better understand the outcomes of adopting these technologies. 

## 5. Conclusions

PGE-1 analogue administration and MP are emerging but understudied strategies to decrease IRI during LT in patients with HCC. Although improved post-transplant RFS is noted, an apparent effect on overall survival has not yet been demonstrated. These findings warrant further prospective studies to fully appreciate the benefit of such interventions in patients undergoing transplantation. 

## Figures and Tables

**Table 1:** Characteristics of the included studies.

Author	Year	Country	Study Design	Recipient Transplant Criteria	Donor Type	Sample Size	Intervention to Decrease IRI	Perfusate Solution	Outcomes Assessed
Kornberg et al. [14]	2015	Germany	Single-centre, retrospective cohort	Milan	NR	106 patients 59 intervention 47 control	Prostaglandin analogue (Alprostadil)	-	Overall survival Recurrence-free survival
Mueller et al. [15]	2020	Switzerland UK	Multi-centre, retrospective cohort	Milan UCSF Metroticket 2.0	DBD DCD	280 patients 70 intervention 210 control	Hypothermic machine perfusion	-	Recurrence-free survival Peri-operative complications Graft and patient survival Biopsy-proven acute rejection De novo tumour growth
Tang et al. [16]	2021	China	Single-centre, retrospective cohort	Milan	DBD	226 patients 30 intervention 196 control	Normothermic machine perfusion	The perfusate contained approximately 1.3 L cross-matched leucocyte-depleted washed red cells, 1.4 L Succinylated gelatinor, 30 mL 5% sodium bicarbonate, 0.5 g metronidazole, 37,500 U heparin, 1.5 g cefoperazone sodium and sulbactam sodium, 30 mL 10% calcium gluconate, 3 mL 25% magnesium sulfate and 250 mL compound amino acid injection.	Overall survival Recurrence-free survival
Rigo et al. [17]	2023	Italy	Single-centre, retrospective cohort	Metroticket 2.0	DBD DCD	326 patients 80 intervention 246 control	Hypothermic machine perfusion	Belzer MP solution (BridgeToLife, Northbrook, IL, USA)	Recurrence-free survival Peri-operative complications Biopsy-proven acute rejection

**Table 2:** Peri-operative and oncologic outcomes of strategies to decrease ischemia-reperfusion injury within the included studies.

Author	Ischemia-Reperfusion Injury	Overall Survival	Recurrence-Free Survival
Kornberg et al. [14]	Significantly lower mean AST peak level post-transplant 581.7 vs. 780.7 IU/mL Significantly lower mean ALT peak level post-transplant 559.6 vs. 701.4 IU/mL Significantly lower mean CRP peak level post-transplant 3.2 vs. 4.6 mg/dL	No significant difference with Alprostadil therapy 91.5% vs. 74.5% at 3 years 82.8% vs. 65.7% at 5 years	Significantly higher with Alprostadil therapy 87.9% vs. 65.3% at 3 years 85.7% vs. 63.1% at 5 years Significantly higher within Milan-Out subgroup of patients HR (95%CI) = 5.09 (1.64–15.76)
Mueller et al. [15]	Significantly higher median ALT day 1 level post-transplant 1305 vs. 893 U/L Significantly lower median CRP day level post-transplant 31 vs. 39 mg/L	NR	Significantly higher with hypothermic machine perfusion compared to untreated DBD liver recipients 92% vs. 73% at 5 years
Tang et al. [16]	Significantly lower ALT day 1 level post-transplant 198.8 vs. 633.8 U/L Significantly lower AST day level post-transplant 437.1 vs. 1571.6 U/L	No significant difference with normothermic machine perfusion 96.7% vs. 90.2% at 1 year 90.6% vs. 68.1% at 3 years	Significantly higher with normothermic machine perfusion 92.2% vs. 73.0% at 1 year 86.7 vs. 46.3% at 3 years Normothermic machine perfusion independently associated with improved recurrence-free survival HR (95%CI) = 3.73 (1.17–11.9)
Rigo et al. [17]	Significantly lower AST peak level post-transplant 903.0 vs. 1140.0 Significantly lower ALT peak level post-transplant 496.5 vs. 742.0	Similar estimated 5-year survival probability based on Metroticket 2.0 model for hypothermic perfusion vs. static cold storage grafts 0.9 vs. 0.9	Comparable recurrence rates for hypothermic perfusion vs. static cold storage grafts 10% vs. 9% RFS HR (95%CI) = 1.34 (0.5–3.4)

AST: aspartate aminotransferase, ALT: alanine aminotransferase, CRP: C-reactive protein, HR: Hazard ratio, 95%CI: 95% confidence interval, DCD: donation after circulatory death, NR: not reported.

**Table 3:** Risk of bias assessments for included studies using the Cochrane ROBINS-I tool. ROBINS-I: Risk of bias in non-randomised studies of interventions.

	Confounding	Selection of Participants	Classification of Intervention(s)	Deviation from Intended Intervention(s)	Missing Data	Outcome Measurement	Reported Result	Overall
Kornberg et al. [14]	Moderate	Moderate	Moderate	Moderate	Low	Low	Low	Moderate
Mueller et al. [15]	Moderate	Moderate	Moderate	Low	Low	Low	Low	Moderate
Tang et al. [16]	Moderate	Moderate	Low	Low	Low	Low	Moderate	Moderate
Rigo et al. [17]	Moderate	Low	Low	Low	Low	Low	Low	Moderate

## Data Availability

The original data presented in the study are openly available at https://pubmed.ncbi.nlm.nih.gov/ (accessed on 14 September 2023), https://ovidsp.ovid.com/ (accessed on 14 September 2023), and https://www.embase.com/ (accessed on 14 September 2023).

## Supplementary Materials

The following supporting information can be downloaded at: https://www.mdpi.com/article/10.3390/curroncol31060221/s1, Table S1: Search Strategy.
