# Supplementary material for: Oncologic Outcomes of Interventions to Decrease Allograft Ischemia-Reperfusion Injury within Patients Undergoing Liver Transplantation for Hepatocellular Carcinoma: A Systematic Review
Source: Curr Oncol. 2024 May 21;31(6):2895–906. doi: 10.3390/curroncol31060221 (PMC11202749; doi:10.3390/curroncol31060221)
Supplement: Supplementary file 1 [file curroncol-31-00221-s001.zip › curroncol-2968003-supplementary.pdf]

Table S1. Search Strategy.

Ovid MEDLINE(R) ALL <1946 to September 12, 2023>

1	hepatocellular carcinoma.mp. or exp Carcinoma, Hepatocellular/	147726
2	liver transplantation.mp. or exp Liver Transplantation/	82208
3	1 and 2 9886	
4	extended criteria.mp. or exp Organ Preservation/	10788
5	ischemia reperfusion injury.mp. or exp Reperfusion Injury/	58826
6	normothermic perfusion.mp.	364
7	4 or 5 or 6	68216
8	exp Recurrence/ or exp Neoplasm Recurrence, Local/ or recurrence.mp.	585989
9	3 and 7 and 8	72
10	donation after circulatory death.mp.	1346
11	4 or 5 or 6 or 1069052	
12	3 and 8 and 11	75
13	machine perfusion.mp.	1734
14	11 or 13	69483
15	3 and 8 and 14	75

Embase Classic+Embase <1947 to 2023 September 12>
EBM Reviews - Cochrane Central Register of Controlled Trials <August 2023>

1	hepatocellular carcinoma.mp. [mp=ti, ab, hw, kw, tn, ot, dm, mf, dv, kf, fx, dq, sh]	176564
2	liver transplantation.mp. [mp=ti, ab, hw, kw, tn, ot, dm, mf, dv, kf, fx, dq, sh]	140922
3	1 and 2 17544	
4	extended criteria.mp. [mp=ti, ab, hw, kw, tn, ot, dm, mf, dv, kf, fx, dq, sh]	2265
5	reperfusion injury.mp. [mp=ti, ab, hw, kw, tn, ot, dm, mf, dv, kf, fx, dq, sh]	87765
6	normothermic perfusion.mp. [mp=ti, ab, hw, kw, tn, ot, dm, mf, dv, kf, fx, dq, sh]	718
7	donation after circulatory death.mp. [mp=ti, ab, hw, kw, tn, ot, dm, mf, dv, kf, fx, dq, sh]	2421
8	machine perfusion.mp. [mp=ti, ab, hw, kw, tn, ot, dm, mf, dv, kf, fx, dq, sh]	3341
9	4 or 5 or 6 or 7 or 8	94199
10	recurrence.mp. [mp=ti, ab, hw, kw, tn, ot, dm, mf, dv, kf, fx, dq, sh]	844780
11	3 and 9 and 10	148
